# Revisits within 48 Hours to a Thai Emergency Department

**DOI:** 10.1155/2016/8983573

**Published:** 2016-07-13

**Authors:** Jiraporn Sri-on, Adisak Nithimathachoke, Gregory Philip Tirrell, Sataporn Surawongwattana, Shan Woo Liu

**Affiliations:** ^1^Emergency Department, Massachusetts General Hospital, Five Emerson, Suite 155, Boston, MA 02114, USA; ^2^Emergency Department, Vajira Hospital, Navamindradhiraj University, 681 Samsen Road, Dusit District, Bangkok 10300, Thailand; ^3^Emergency Department, Massachusetts General Hospital, 55 Fruit Street, Zero Emerson Place, Room 346, Boston, MA 02114, USA

## Abstract

*Objective*. Emergency department (ED) revisits are a common ED quality measure. This study was undertaken to ascertain the contributing factors of revisits within 48 hours to a Thai ED and to explore physician-related, illness-related, and patient-related factors behind those revisits.* Methods*. This study was a chart review from one tertiary care, urban Thai hospital from October 1, 2009, to September 31, 2010. We identified patients who returned to the ED within 48 hours for the same or related complaints after their initial discharge. Three physicians classified revisit as physician-related, illness-related, and patient-related factors.* Results*. Our study included 172 ED patients' charts. 86/172 (50%) were male and the mean age was 38 ± 5.6 (SD) years. The ED revisits contributing factors were physician-related factors [86/172 (50.0%)], illness-related factors [61/172 (35.5%)], and patient-related factor [25/172 (14.5%)], respectively. Among revisits classified as physician-related factors, 40/86 (46.5%) revisits were due to misdiagnosis and 36/86 (41.9%) were due to suboptimal management. Abdominal pain [27/86 (31.4%)] was the majority of physician-related chief complaints, followed by fever [16/86 (18.6%)] and dyspnea [15/86 (17.4%)].* Conclusion*. Misdiagnosis and suboptimal management contributed to half of the 48-hour repeat ED visits in this Thai hospital.

## 1. Introduction

One measure of quality of care is the emergency department (ED) revisit. Revisits within three visits have been associated with an increased long-term mortality rate (hazard ratio 1.89) [1]. Screening tools for monitoring and auditing patients who return early to the ED are becoming increasingly necessary to improve quality of care [2–4]. While there is not a uniform definition of acute revisits, revisits are most often defined as visits within 24–72 hrs [2, 5–7]. The rate of 24–72-hour ED revisitation by discharged patients can range within 0.39–4.9% [5–10] and is influenced by patient-related and illness-related factors [8, 11, 12]. The main reason for revisits in US hospitals was patient-related factors (53%), whereas illness-related factors were cited as the main reason for revisits for Taiwan hospitals and New Zealand hospitals (61%), respectively. One-third of return visits are avoidable [5, 6].

Among elderly patients, advanced age, cognitive impairment, male gender, living alone, and dyspnea as a chief complaint increased the risk of revisits [13, 14]. Atypical presentations of common diseases such as vascular, neurologic, and infectious diseases may result in misdiagnosis or premature discharge from the ED and, consequently, a return visit shortly after being seen [15–17].

The literature has largely focused on quality of care in high income countries [4, 8, 11, 12], in which EDs have ready access to their hospital's full range of diagnostics. This area is relatively unexplored in middle income countries which may not have the same instruments available for investigation; hence, revisit rate may be increasing. Thailand is now a middle income country with an advancing healthcare system. One study in Thailand examined revisits but focused on chief complaint and not the reasons behind the revisits [18]. No study has examined the factors associated with ED revisits in Thailand. The purpose of this study was to ascertain factors associated with 48-hour Thai ED revisits.

## 2. Methods

This study was a chart review at one tertiary care, 700-bed, urban, and teaching hospital in Bangkok, Thailand, from October 1, 2009, to September 31, 2010. The ED has an annual patient volume of 80,000 patients. Two emergency physicians (EPs) and two residents work during the morning shift, one EP and two residents work during evening shift, and one EP and one resident work during night shift. Two residents from other specialties are available for the treatment of nonurgent patients during the evening shift (16:00–24:00). All doctors who have a Thai medical license have the authority to discharge patients themselves.

We included patients who returned to the ED within 48 hours for the same or related complaints after their initial discharge. We used the 48-hour revisit rate according to our hospital quality assurance (QA) indicator. We excluded patients who had outpatient physician appointments or patients who returned with a different medical problem unrelated to the initial visit. The EP who was in charge on that day flagged the chart of any patients who had been seen within the previous 48 hours. At the time of this study, the hospital was transitioning from paper records to a computerized system. Subsequently, medical data were recorded either manually or digitally.

We collected data on age, gender, means of arrival, triage level, arrival and discharge time, health insurance, chief complaint, diagnosis, and type of disposition.

In Thailand, there is a public health insurance option called “the 30-baht healthcare scheme” in which patients must register at the closest hospital to their residence for regular medical care. They may receive emergency care at any hospital if the receiving ED doctor determines it is a true emergency, but if they seek care for what the doctor determines not to be a true emergency at a hospital other than the one to which they are registered, they are responsible for the full cost of their care (30 baht or approximately $1 US dollar).

Contributing factors for patient revisits were determined to be one of three categories: physician-related factors, illness-related factors, and patient-related factors which we adapted from the methods of Wu et al. [10, 11]. Three emergency physicians agreed on the definitions of each of the categories and were trained on sample patients charts before data collection. One ED resident and one ED attending physician independently classified patient revisit records into one of the three categories:

1. Illness-related factors include
  - patients receiving appropriate emergency medical care but there being either disease progression, failure of standard care to improve symptoms, or recurrence of symptoms,
  - atypical presentation of disease.
2. Patient-related factors include
  - patients who revisited the ED due to anxiety over their illness when symptoms had not worsened or improved, patients who had psychological problems such as substance abuse, or patient noncompliance,
  - patients who left the ED against medical advice.
3. Physician-related factors include the following:
  - Suboptimal treatment: the physician made the correct diagnosis but made an error during the course of treatment (not giving proper analgesic or antibiotic, wrong drug or treatment being given, or adverse drug reaction). For example, patients with chronic obstructive pulmonary disease with an exacerbation received only *β* agonists but not steroids.
  - Misdiagnosis: the diagnosis made by the first physician was incorrect as determined from the chart review. For example, patients with abdominal pain and diarrhea initially were diagnosed with gastroenteritis and discharged home but returned and were correctly diagnosed with appendicitis.
  - Inappropriate discharge instructions: patients left the ED without receiving discharge instructions or without arranging appropriate follow-up with the doctor. For example, children with fever and no toxic signs returned within 12 hours for ongoing fever.

In the case of inconsistent categorization among the two reviewers, the discrepancy was reviewed by one blinded ED attending physician before being reassigned to a category. All data was recorded and analyzed using Microsoft Excel 2007. Interrater reliability was calculated using Kappa statistic. Categorical data was presented as percentages. Continuous data was recorded as a mean with standard deviation if normally distributed or a median with interquartile range if nonnormally distributed. This study was approved by our hospital Institutional Review Board.

## 3. Results

Between October 1, 2009, and September 31, 2010, 88,342 patients presented to the ED, and 172 of these patients returned to ED within 48 hours. Their mean age was 38 ± 5.6 (SD) years, and 86/172 (50%) were male. All patients were of Asian descent. Only 6 (3.5%) patients were transported by ambulance. The 30-baht healthcare scheme represented the majority of payment methods (Table 1).

The unscheduled revisit rate was 0.19% (172 visits/88,342 patients). Over a third of patient revisits occurred during the evening shift (37.8%, 65/172). However, when compared with the number of patients the night shift had the highest revisit rate (0.30%, 48 visits/15,906 patients), while the evening shift had the lowest rate (0.16%, 65 visits/39,559 patients) (Table 2).

Physician-related factors account for 86 (50.0%) of the ED revisits. Illness-related factors accounted for 61 (35.5%) revisits and patient-related factors accounted for 25 (14.5%) revisits. Misdiagnosis was the most common reason for patient revisits among physician-related factors with 40 (46.5%) revisits, followed by suboptimal management with 36 (41.9%) revisits and inappropriate discharge advice and appointment scheduling with 10 (11.6%) revisits (Table 3).

For patients who revisited the ED for physician-related factors, abdominal pain was the most common chief complaint as regards misdiagnosis (18.6% revisits) while dyspnea was the main chief complaint for suboptimal management (13.9% revisits) and fever was the main chief complaint for inappropriate discharge advice (5.8% revisits) (Tables 4 and 5). Kappa was 0.74 (95% confidence interval: [0.65 to 0.83]) for interrater reliability.

## 4. Discussion

In our study, physician-related factors were responsible for 50% of the revisits; this rate was higher than data reported from Pierce et al. [8] and Wu et al. [10]. Pierce et al. found that only 18% of all unscheduled revisits were the result of physician-related factors, while Wu et al. reported 11% of revisits were due to physician-related factors.

Misdiagnosis was the most common type of physician-related factor for revisits in our study, accounting for 46.5% of physician-related revisits. The rate of misdiagnosis differs greatly throughout the literature and ranges within 5.7–9.0% [10–12]. One possible source of misdiagnosis is early discharge of the patient after their symptoms improve, but without complete and exhaustive investigation of the patient for a definitive diagnosis. As ED and hospital crowding increases [19], a greater demand for beds than hospital capacity may pressure emergency physicians to discharge a patient. The increasing number of patients staying in observation rooms in ED further tax the amount of time emergency physicians and nurses have available to see new and revisiting emergency patients who may need more attention, a situation which may decrease the efficiency of emergency treatment [5].

Abdominal pain was the most common chief complaint for misdiagnosed patients in this study. The clinical presentation of abdominal pain is always challenging for EPs. Clinical decision making alone is not helpful for all patients, especially for elderly patients [20–22]. The rate of correct diagnosis for abdominal pain varies from 40% to 82% [21, 22]. Utilization of abdominal computer tomography (CT) has greatly benefitted patients and physicians in the investigation of this complaint [21, 23, 24]. The lack of availability of CT scans for the investigation of this complaint during the night shift in the ED in this hospital may be one reason for the high rate of misdiagnosis of abdominal pain. Suboptimal management accounted for approximately one-third of physician-related factors.

Improving physician knowledge and skills for optimal management will help avoid redundant or unneeded use of ED diagnostics and resources. Data shows that suboptimal information sharing was associated with increased odds of an ED return visit [25]. ED staff should improve discharge instructions to decrease revisit rates. Some of the discharged patients received paper discharge instructions but they may not have had an adequate understanding of the instructions that were given. Ensuring patients understand their discharge instructions should be confirmed before they leave the ED.

Illness-related factors were responsible for 35.4% of the revisits; this rate was lower than reported by the Wu et al. [11] and Hung et al. [26] studies. Wu et al. [11] reported 80.9% of unscheduled revisits were related to illness factors. Hung et al. [26] found that over half of the revisits by patients were associated with recurrent disease processes (60.4%). In our study, recurrent symptoms, disease progression, and lack of symptom improvement were the major reasons for revisits among illness-related factors. Only 1.7% had an atypical presentation of the disease. Hu [5] reported the most common diseases for unavoidable revisits were chronic obstructive pulmonary disease (COPD), benign prostate hypertrophy, urolithiasis, bronchial asthma, and coronary artery disease. Some chronic diseases such as COPD have the potential for recurrent attacks or end stage malignancy which may require pain control. Hence, their revisit may be classified as either disease progression or recurrent symptoms.

Patient-related factors comprised 14.5% of revisits, which was comparable to the findings in other studies. Wu et al. [11] reported 10.9% and Hung et al. [26] reported 14.2% of revisits were due to patient-related factors. The main reason for patient-related revisits in our study was an overanxious reaction; for example, patients returned within 48 hours for the same reason when their symptoms had not worsened. In contrast to our study, Wu et al. reported patients returning after initially leaving the ED against medical advice were the primary factor.

The rates of 48 hrs unscheduled return visits were 0.19%, which is lower than rates reported in other studies [5–12]. There are multiple explanations of why our revisit rate is low. First, our hospital used paper records at the time, and an error may have occurred if the EP did not review the record from the patient's previous visit. Second, patients who returned during the morning shift with nonurgent conditions are sent directly to the general practitioner outpatient department (OPD) which may have resulted in a lower revisit rate than actually occurred. Third, as a consequence of the policy of the Thai national health insurance system, patients are more likely to go to the hospital at which they are registered for a return visit because they do not have to pay for the incurred medical expense. This would not register as a revisit if they initially sought emergency care at another hospital. However, doctors may advise patients to go to the hospital that they are registered at if their symptoms do not improve.

## 5. Limitations

This study has several important limitations, including the inherent limitations of a chart review to accurately measure quality of care. There may be a lack of documentation regarding why a particular treatment was not given; for example, if a provider was concerned about infection, they may have not ordered steroids for a moderate asthma attack. In addition, our charts were not reviewed by an outside reviewer. Subjective bias may have therefore occurred in the evaluation of revisit causes. Our reviewers strived to judge the reasons for return visits objectively, but there exists the possibility of some level of subjective bias. Additionally, we could not include patients who went to another hospital for a revisit for the same complaint, after initially being treated at our hospital. The transition of system design from manual to computer records potentially unreported revisits. Any study that is only performed at one site is inherently limited in its ability to generalize findings among a wider population.

## 6. Conclusion

Misdiagnosis and suboptimal management contributed to half of the repeat visits of ED patients seen within 48 hours. It is important to identify potential areas for improvement in the quality of care among revisit patients. The implementation of a policy to digitally monitor for revisits may help physicians learn common characteristics among revisiting patients and subsequently alter their plan of care for frequently returning chief complaints. Improving the education and knowledge of emergency physicians for making the decision to discharge patients early could also be beneficial. Further study is warranted into risk factors for misdiagnoses and ways to improve quality of care in the ED.

## Figures and Tables

**Table 1:** Demographics of revisiting patients, *n* = 172.

Variable	*n* (%)
Gender	
Male	86 (50)
Age	
<15	32 (18.6)
15–30	42 (24.4)
31–45	36 (20.9)
46–60	21 (12.2)
>60	41 (23.8)
Mode of arrival	
Nonambulance	166 (96.5)
Ambulance	6 (3.5)
Insurance	
Private insurance	1 (0.6)
Social security insurance	30 (17.4)
The 30-baht healthcare scheme	57 (33.1)
Government employee	28 (16.3)
Self-pay	56 (32.6)

**Table 2:** Visit characteristics of initial emergency department visit, *n* = 172.

	*n* (%)	ED patients	Rate
Revisit	172 (100)	88,342	0.19
ED shift			
Night (0.00–8.00)	48 (27.9)	15,906	0.31
Morning (8.01–16.00)	59 (34.3)	32,877	0.18
Evening (16.01–24.00)	65 (37.8)	39,559	0.16
Triage level			
Nonurgent	38 (22.1)	53,745	0.07
Urgent	131 (76.2)	31,299	0.42
Emergency	3 (1.7)	2,545	0.11
Resuscitation	0	753	0

**Table 3:** The contributing factors for revisits.

	*n* = 172 (%)
Patient-related factors	25 (14.5)
Against advice	6 (3.5)
Overanxious reaction	19 (11.0)
Illness-related factors	61 (35.5)
Atypical presentation of disease	3 (1.7)
Recurrent symptom, no improvement, or disease progression	58 (33.7)
Physician-related factors	86 (50.0)
Misdiagnosis	40 (23.3)
Suboptimal management	36 (20.9)
Inappropriate discharge advice or appointment	10 (5.8)

**Table 4:** Most common chief complaints from physician-related factors, *n* = 86 (%).

	Misdiagnosis	Suboptimal management	Inappropriate discharge advice
	*n* = 40 (%)	*n* = 36 (%)	*n* = 10 (%)
Abdominal pain	16 (18.6)	10 (11.6)	1 (1.2)
Fever	3 (3.5)	8 (9.3)	5 (5.8)
Dyspnea	2 (2.3)	12 (13.9)	1 (1.2)
Headache	5 (5.8)	4 (4.6)	2 (2.3)
Dizziness	4 (4.6)	2 (2.3)	1 (1.2)
Chest pain	4 (4.6)		
Others	6 (6.9)		

**Table 5:** Final disposition (second visit).

Final disposition (second visit)	*n* (%)
ED observe unit	64 (37.2)
Admit to ward	57 (33.1)
Admit to ICU	3 (1.7)
Discharge	48 (27.9)
